# Kidney allograft infarction associated with transplant renal artery stenosis in a COVID-19 kidney transplant recipient 

**DOI:** 10.5414/CNCS110462

**Published:** 2021-07-26

**Authors:** Ekamol Tantisattamo, Donald C. Dafoe, Antoney J. Ferrey, Hirohito Ichii, Richard A. Lee, Jonathan E. Zuckerman, Anthony E. JR. Sisk, Ted Farzaneh, Jack Guccione, Nii-Kabu Kabutey, Kamyar Kalantar-Zadeh, Uttam G. Reddy

**Affiliations:** 1 Harold Simmons Center for Kidney Disease Research and Epidemiology, Division of Nephrology, Hypertension and Kidney Transplantation, Department of Medicine, University of California Irvine School of Medicine, Orange,; 2 Nephrology Section, Department of Medicine, Tibor Rubin Veterans Affairs Medical Center, Veterans Affairs Long Beach Healthcare System, Long Beach, CA,; 3 Multi-Organ Transplant Center, Section of Nephrology, Department of Internal Medicine, William Beaumont Hospital, Oakland University William Beaumont School of Medicine, Royal Oak, MI,; 4 Division of Kidney and Pancreas Transplantation, Department of Surgery,; 5 Division of Pulmonary Diseases and Critical Care Medicine, Department of Medicine, University of California Irvine School of Medicine, Orange,; 6 Department of Pathology and Laboratory Medicine, David Geffen School of Medicine at University of California Los Angeles, Los Angeles,; 7 Department of Pathology and Laboratory Medicine, University of California, Irvine,; 8 Division of Division of Vascular and Endovascular Surgery, Department of Surgery, University of California Irvine School of Medicine, Orange, and; 9 Lundquist Biomedical Research Institute at Harbor-UCLA Medical Center, Torrance, CA, USA

**Keywords:** coronavirus disease 2019, COVID-19, kidney allograft infarction, kidney transplantation, SARS-CoV-2, severe acute respiratory syndrome coronavirus 2, thromboembolism, thrombosis, transplant renal artery stenosis

## Abstract

Kidney allograft infarction is rare, but an urgent condition that requires prompt intervention to avoid allograft loss. Renal artery thrombosis is the leading cause of infarction. Apart from traditional risk factors for thrombosis, emerging SARS-CoV-2 predisposes patients to thrombotic diseases both in arterial and venous vasculatures. We report a case of kidney transplant recipient with known transplant renal artery stenosis (TRAS) status post angioplasty with severe COVID-19, complicated by oliguric acute kidney injury requiring continuous renal replacement therapy (CRRT). She did not have a history of thromboembolic disease. The hospital course was complicated by new-onset atrial and ventricular fibrillation and cardiac arrest requiring multiple rounds of cardiopulmonary resuscitation. She had no signs of renal recovery, and an abdominal CT scan showed evidence of allograft infarcts. She underwent an allograft nephrectomy. Pathology revealed diffuse thrombotic microangiopathy involving glomeruli, arterioles, and arteries associated with diffuse cortical infarction with negative SARS-CoV-2 immunostain and in situ hybridization. This is the first case of kidney allograft infarct with a history of TRAS in a COVID-19 patient. Underlying TRAS and COVID-19-associated thrombosis in this patient are unique and likely play a key role in allograft infarction from arterial thrombosis. Recognizing risk factors and early therapy for allograft infarction may improve transplant outcomes.

## Introduction 

Over the past year, since the first confirmed coronavirus disease 19 (COVID-19) cases were reported in December 2019, the number of cases continues to increase with a broad spectrum of clinical presentations and complications. An increase of thrombotic and thromboembolic complications has been associated with this emerging severe acute respiratory syndrome coronavirus 2 (SARS-CoV-2) in both arterial [1] and venous [2] systems. 

Kidney infarction is commonly related to thromboembolic diseases or injury to the renal vasculatures. Thus far, there has been one reported COVID-19 case who presented with acute kidney allograft infarction diagnosed by imaging [3]. Since an underlying vascular dysfunction may contribute to allograft thrombosis, we report a case of kidney transplant recipient with a history of transplant renal artery stenosis (TRAS) who presented with severe COVID-19 and developed allograft infarction from arterial thrombosis requiring allograft nephrectomy. 

We discuss the pathogenesis of allograft infarction in the setting of COVID-19 and the history of TRAS. Several biomarkers related to the risk of thrombosis and outcomes in COVID-19 and allograft imaging may detect early and evolving thrombosis. 

## Case history 

A 33-year-old obese woman with end-stage kidney disease (ESKD) secondary to type 1 diabetes presented with fever, cough, and bloody diarrhea 1 day prior to admission (PTA). 

**Figure 1.**
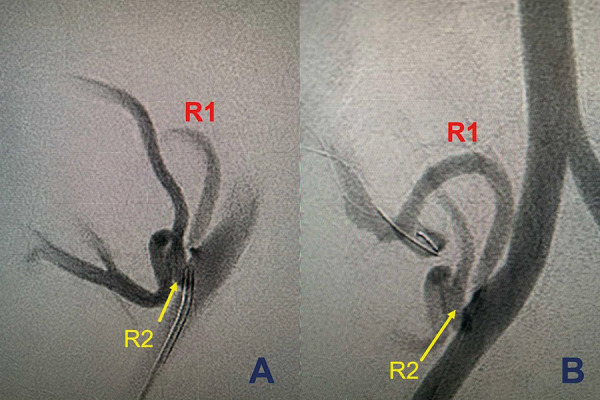
Carbon dioxide angiography performed at 4.5 months post-transplant demonstrates two transplant renal arteries (R1 and R2) which were 4 mm apart on the donor aortic patch. The caudal renal artery (R2) was divided into two branches. A: A high-grade stenosis or kink at the level just distal to the anastomosis of the cephalad renal artery (R1) and wide patent of the caudal renal artery (R2). B: A marked improvement and more brisk flow through to cephalad renal artery (R1) into the kidney allograft after a 2.5 mm × 2 cm Ultraverse balloon angioplasty at the level of the stenosis.

Approximately 6.5 months PTA, she underwent a 0-A-B-DR-mismatched antigen deceased donor kidney transplantation (DDKT) with antithymocyte globulin induction. At 4.5 months post transplant, she developed acute kidney injury (AKI) with serum creatinine (SCr) of 5.1 mg/dL from the baseline of 0.9 – 1.1 mg/dL secondary to TRAS of 1 of 2 transplant renal arteries, which were 4 mm apart on the donor aortic patch. Only cephalad renal artery with high-grade stenosis at the level just distal to the anastomosis was successfully intervened by CO_2_ balloon angioplasty and the caudal renal artery was wide patent (Figure 1). Follow-up SCr was improved to a new baseline of 1.5 – 1.7 mg/dL. Clopidogrel was started. Maintenance immunosuppression included cyclosporine, mycophenolate sodium (MPS), and prednisone. 

Five days PTA, she experienced headaches, cough, and back pain. Three days later, she started having a fever with a temperature (T) of 38.3 C, body aches, and nasal congestion. Due to the concern for possible COVID-19, a nasal-pharyngeal swab for SARS-CoV-2 by a real-time reverse transcription polymerase chain reaction (rtPCR) test was obtained on the next day. 

On the day of admission, she presented with bloody diarrhea, nausea, and vomiting. The COVID-19 test had returned positive, and she was advised to go to the emergency room. Her T was 38.3 ^○^C, heart rate (HR) 90 beats/minute, respiratory rate (RR) 18/minutes, and blood pressure (BP) 178/77 mmHg. SpO_2_ was 93% on room air. Venous blood gas revealed pH 7.2, PvCO_2_ 37.2 mmHg, PvO_2_ 38.8 mmHg, SvO_2_ 68.4%, and total CO_2_ 18 mmol/L. She developed oliguric AKI with an initial SCr of 4.2 mg/dL and peak at 5.9 mg/dL on the next day. Complete blood count (CBC) showed hemoglobin of 8.2 g/dL, white blood cells (WBC) 3,900/µL, and platelet 163,000/µL, and there was no evidence of hemolysis. Her mental status was intact. Transplant renal ultrasound with color Doppler (U/S) showed resistive indices of 0.4 – 0.46, normal intrarenal waveforms with the appropriate diastolic flow, peak systolic velocities (PSV) of the first and second renal arteries of 86 – 158 and 64 – 81 cm/s, respectively, and no renal artery stenosis on color Doppler imaging. The external iliac artery PSV was 141 and 126 cm/s superior and inferior to the anastomosis, respectively. The transplant renal vein and external iliac vein were patent. There was no evidence of hydronephrosis. Clopidogrel was discontinued due to gastrointestinal (GI) bleeding. 

On hospital day (HD) 2, she developed septic shock requiring norepinephrine and subsequently additional phenylephrine. She was empirically treated with levofloxacin and ceftriaxone. She was intubated for acute hypoxemic respiratory failure. Given the worsening metabolic acidosis, continuous renal replacement therapy (CRRT) was initiated. She had oliguria since admission with the maximum urine output of 190 mL/24 hours on HD 3, and then she became anuric. A repeated U/S on HD 4 to assess vascular flow revealed normal vascular findings and no evidence for hydronephrosis. 

MPS was held, and then cyclosporine was discontinued. She had elevated inflammatory markers (Table 1). Dexamethasone was started. She received remdesivir followed by convalescent plasma. 

On HD 7, a chest computed tomography (CT) scan showed bilateral diffuse ground-glass opacities but no evidence of pulmonary embolism. Empiric heparin drip was started for the high-risk hypercoagulable state. Apart from positive sputum cultures on HD 3 and 8, which grew 2+ group B β-hemolytic streptococcus and few *Staphylococcus aureus*, respectively, bacterial and fungal blood cultures were negative. Urinalysis showed 3 WBC/HPF, 1 RBC/HPF, 1 squamous epithelial cell, and few bacteria, which did not meet the criteria for a reflex culture. Broad-spectrum empiric antibiotics including meropenem, vancomycin, and micafungin were initiated. 

**Figure 2.**
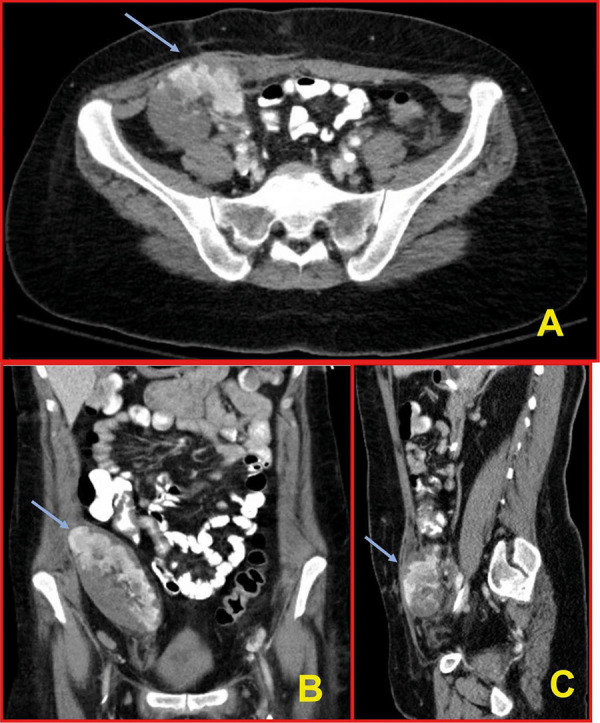
Abdomen and pelvis CT scan with contrast on hospital day 9 revealed abnormal, heterogeneous parenchymal enhancement of the transplant kidney allograft with multifocal, peripherally located areas of nonenhancement with an appearance suggestive of multiple renal infarcts (blue arrows). No hydronephrosis (1A: cross-sectional view, 1B: coronal view, and 1C: sagittal view).

On HD 9, an abdominal and pelvic CT scan demonstrated no intra-abdominal source of infection; however, it incidentally revealed multiple allograft infarcts (Figure 2). 

**Figure 3.**
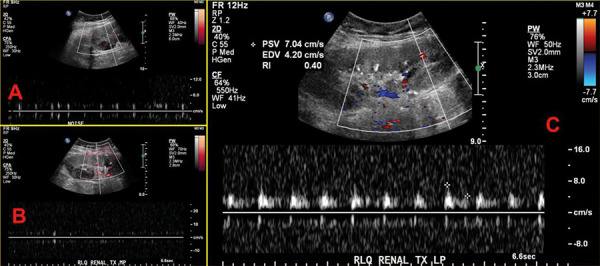
Transplant kidney ultrasound with color Doppler duplex on hospital day 13 revealed no flow detected in the upper and mid poles of transplant kidney allograft. Lower pole parenchyma demonstrates low resistive waveform, which may be related to ischemia (2A: upper pole, 2B: mid pole, and 2C: lower pole).

On HD 10, she developed new-onset atrial fibrillation (AF). She then developed ventricular fibrillation (VF) and 4 episodes of serial cardiac arrest. She achieved a return of spontaneous circulation after cardiopulmonary resuscitation (CPR) and a normal sinus rhythm. U/S showed no flow in the mid and upper pole of allograft and low resistive waveform in the lower pole parenchyma (Figure 3). A hypercoagulable workup was negative except for slightly elevated homocysteinemia (Table 1). 

**Figure 4.**
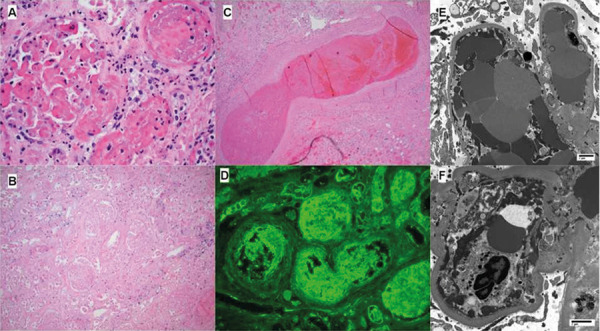
Figure 4. Explanted allograft kidney demonstrated diffuse thrombotic microangiopathy (TMA) with extensive cortical infarction. (A) Extensive glomerular, arteriolar, and interlobular artery thrombosis (B) Extensive cortical necrosis (C) Interlobar artery thrombosis (H & E stained sections). (D) Fibrinogen staining of arterial thrombosis. (E, F) Electron micrographs of glomerular capillary loops involved by thrombosis. Dysmorphic red blood cells and fibrin tactoids distend capillary loops. Endothelial cells are denuded from basement membranes. Podocyte foot processes are detached. Light microscopy and immunofluorescence microscopy images are × 400.

On HD 16, she underwent an allograft nephrectomy, as the infarcted allograft was a possible source of infection. Maintenance hemodialysis was initiated. The allograft was pale and small consistent with arterial thrombosis. Pathology demonstrated diffuse thrombotic microangiopathy (TMA) involving glomeruli, arterioles, and arteries with diffuse infarction of the renal parenchyma. C4d staining was negative and there was no pathological evidence of acute cellular or antibody-mediated rejections. SARS-CoV-2 immunostain and in situ hybridization (ISH) were negative. No viral particles were present in any compartment (Figure 4). There was a donor-specific class II human leukocyte antigen (HLA) antibody to DR53, but no donor-specific class I HLA antibody. 

**Figure 5.**
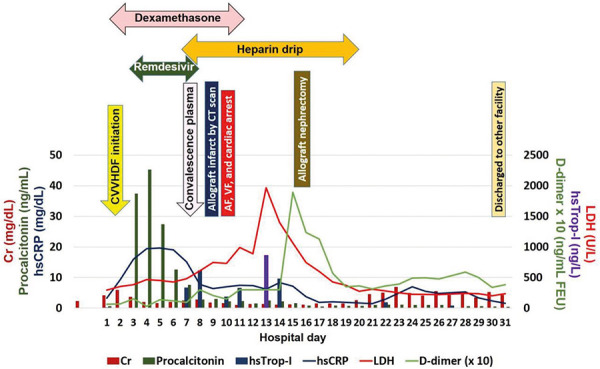
Clinical course during hospitalization relating to different biomarkers. AF = atrial fibrillation; Cr = serum creatinine; CVVHDF = continuous venovenous hemodiafiltration; hsCRP = high-sensitive c-reactive protein; hsTrop-I = high-sensitive Troponin I; LDH = lactate dehydrogenase; VF = ventricular fibrillation.

On HD 31, she was transferred to another facility for a lower level of care. Clinical course and biomarkers indicating thrombotic risk and inflammation related to COVID-19 are shown in Figure 5. 

## Discussion 

Our patient presented with COVID-19 complicated by multiple severe complications including septic shock, VF cardiac arrests, and unsalvageable allograft infarction. 

Etiologies of AKI at the initial presentation could be multifactorial including prerenal causes due to diarrhea and GI bleeding. Her fever, bloody diarrhea, and AKI with diffuse TMA in the allograft might raise the concern for the typical hemolytic uremic syndrome (HUS) classically from enterohemorrhagic *Escherichia coli*. However, she had no signs of hemolysis or thrombocytopenia and clopidogrel could increase the risk of GI bleeding, particularly in the setting of septic shock. With the presence of the donor-specific class II HLA antibody, she was at risk for acute antibody-mediated rejection (ABMR) which may present with TMA demonstrated in the pathological findings of the explanted kidney allograft. However, there were no C4d or other typical pathological features of ABMR. Ischemic acute tubular necrosis secondary to hemodynamic instability from septic shock was likely an initial cause of AKI. The new-onset AF, and then VF, and 4 episodes of serial cardiac arrest on HD 10 caused additional insults for AKI. The lack of SARS-CoV-2 immunostain and negative ISH, as well as no viral particles in any compartment of the explanted allograft histology, make SARS-CoV-2 unlikely to be the direct cause of AKI. However, given its association with a hypercoagulable state, COVID-19 was possibly the main cause of allograft infarction leading to severe AKI and subsequently allograft loss in our patient. 

Two of the most common causes of kidney infarction are a thromboembolic phenomenon and in situ thrombosis. The pathogenesis of the allograft infarction in the setting of COVID-19 in our patient consisted of endothelial injury and arterial thrombosis, but an unlikely thromboembolic event. 

Endothelial injury of the transplant renal arteries is likely the main contributing factor. SARS-CoV-2 infects cells via angiotensin-converting enzyme 2 which is expressed on the surface of various cells in organs including endothelial cells and this facilitates endotheliitis [4]. Although the autopsy case series reported direct SARS-CoV-2 cytotoxicity involving renal tubular epithelium [5], negative SARS-CoV-2 in our patient’s pathology may suggest systemic procoagulation effect of SARS-CoV-2 beyond direct viral cytotoxicity. 

Endothelial dysfunction is associated with a procoagulation state [6], which increases the risk of arterial thrombosis. The pathology findings confirmed that arterial thrombosis played a role in the allograft infarction of our patient. There are several underlying risk factors and precipitating causes of thrombosis in this patient. In addition to several traditional risk factors for arterial thrombosis including diabetes, dyslipidemia, hypertension, obesity, and elevated homocysteinemia [7], COVID-19 may contribute to an increased risk of arterial thrombosis not only due to endothelial injury and increased inflammation but also platelet activation and stasis [8]. Underlying immunosuppressive milieu, particularly associated with calcineurin inhibitors, increases the risk for endothelial dysfunction [9] and TRAS. However, immune-mediated endothelial damage is less likely to be the cause of TRAS in our patient who was diagnosed with TRAS at 2 months post transplant since the immunological process causing endothelial injury likely occurs at the late post-transplant period [10]. Oxidative inflammatory response secondary to allograft ischemic reperfusion injury after CPR can activate procoagulation cascade [11]. Moreover, impaired oxygen delivery to the allograft from hypoxemia and the COVID-19-related inflammatory cascade [12], which can cause hypercoagulable state, microangiopathy, and transplant renal artery thrombosis [13], can further worsen ischemic injury and the circulation to the allograft and potentially promote allograft infarction. High-dose steroids that the patient received for septic shock can activate the tissue factor/factor V pathway [14]. Although hypotension during CPR appeared to contribute to allograft thrombosis from sudden hemodynamic change and could increase thrombotic risk by potentiating platelet aggregation and vasoconstriction in the setting of norepinephrine administration [15], the CT scan revealed evidence of allograft infarction 1 day prior to cardiac arrest. Therefore, a hypercoagulable state rather than hypotension was likely the main cause of allograft thrombosis in this patient. 

Allograft infarction was detected by the CT scan on HD 9 which is close to the known duration for a full restoration of adenosine diphosphate-induced platelet responses of 7 days after clopidogrel discontinuation [16]. Although discontinuation of clopidogrel on the day of admission may increase the risk of thrombosis potentially leading to the restenosis of previous TRAS, U/S did not reveal evidence of hemodynamic significance to suggest the restenosis. Moreover, the allograft had 2 renal arteries that were 4 mm apart on the donor aortic patch (Figure 1). The abdominal CT scan revealing multifocal infarcted areas including upper and lower poles of the allograft suggests a systemic prothrombotic effect related to SARS-CoV-2 leading to thrombosis of both arteries rather than the lack of antiplatelet effect on the previously stenotic artery. 

Nevertheless, a combination between prior TRAS and SARS-CoV-2 may theoretically increase the risk for transplant renal arterial thrombosis in our patient. Some in vivo studies in pigs demonstrated a 1-month duration for a total relining of the endothelial surface after the endothelium of a segment of the left anterior descending coronary artery was gently denuded. However, the regenerated endothelium had impairment of the endothelium-dependent relaxation increasing propensity of endothelium-dependent contractions, although the intrinsic ability to produce nitric oxide of the regenerated endothelium was not affected. This evidence demonstrated a selective dysfunction of the regenerated endothelium which remained at least 6 months after endothelial denudation [17, 18, 19, 20]. Since our patient had a successful CO_2_ balloon angioplasty for TRAS of 1 of 2 transplant renal arteries ~ 4.5 months before developing COVID-19, possible impaired endothelial dysfunction and hypercoagulable effect of SARS-CoV-2 likely contribute to acute transplant renal arterial thrombosis. Moreover, calcineurin inhibitor is associated with endothelial dysfunction and this effect was more pronounced in patients taking cyclosporine (CsA), like our patient, compared to those taking tacrolimus [9]. 

An arterial thromboembolic event is a potential factor in her worsening kidney allograft infarction; however, its role as a primary cause of infarction is unclear. The abdominal CT scan incidentally showed several infarcted areas in the allograft 1 day before she developed the new-onset AF, VF, and cardiac arrest. U/S performed 3 days after the onset of AF revealed progression of the infarcted areas. However, the morphologic features of the renal thrombi were not suggestive of emboli, and the diffuse nature of the microvascular thrombosis was also inconsistent with thromboembolic disease. 

Several biomarkers have been associated with thrombotic risks or poorer outcomes, especially mortality, in COVID-19 patients. 

Hazanov et al. [21] reported characteristics of 44 non-transplant patients with acute renal embolism and AF. Up to 14% had prior embolic events, and 93% had an elevated serum lactate dehydrogenase (LDH) > 400 U/dL with the mean of 1,100 ± 985 U/dL. A previously published kidney allograft infarct case with COVID-19 [3] and our patient had no history of AF or thromboembolic event before the allograft infarction was diagnosed. Our patient’s LDH on admission was slightly elevated with the level of 294 U/L, and it had trended up to a peak of 1,967 U/L 3 days after cardiac arrest. A high-sensitive troponin I (hsTrop-I) elevated to 337 ng/L (reference 0 – 15) (Figure 5). Although the elevated LDH can also be seen in acute myocardial infarction (AMI) [21], it is likely explained by allograft infarction in our patient given the clinical presentation and the 12-lead electrocardiogram suggested demand ischemia rather than AMI. 

Moreover, LDH was reported as one of the prognostic biomarkers in COVID-19 patients [22, 23, 24]. A pooled analysis from 9 publications including 1,532 COVID-19 patients showed that elevated LDH > 255 U/L was associated with 6 and 16 times greater odds of severe diseases and mortality, respectively [24]. On the other hand, a decrease in LDH after its elevation was associated with radiological improvement in COVID-19 patients [23]. 

D-dimer is another marker that is related to outcomes in COVID-19 patients. It also correlates in the same direction as other inflammatory markers such as high sensitive C-reactive protein. Compared to bacterial pneumonia, COVID-19 patients had a significantly higher d-dimer, and decreased level was correlated with improved prognosis. However, d-dimer had a low predictive value for thromboembolism [25]. 

Pro-calcitonin, which is generally elevated in bacterial pneumonia, is associated with COVID-19 severity. Although a meta-analysis of observational studies demonstrated an association between elevated pro-calcitonin and severity of COVID-19, this relationship is possibly due to concomitant bacterial infection in severe COVID-19 [26]. 

hsTrop-I is another biomarker that is related to poor outcomes in COVID-19 patients yet is necessary to represent AMI [27]. Additionally, the results from prior studies may be affected by confounding by indication of testing troponin-I in COVID-19 patients who were sicker or at risk for cardiac disease. 

Our patient’s elevated peak LDH after cardiac arrest trended down with improved clinical condition, which was consistent with previous studies [23]. Moreover, LDH, d-dimer, pro-calcitonin, and hsTrop-I increased prior to the diagnosis of allograft infraction as well as had increased and decreased along with worsening and improving clinical course (Figure 5). 

Although there is no current consensus guideline to evaluate the risk of thromboembolism due to COVID-19, especially in the transplant population, these biomarkers may guide clinicians to assess the risk, as well as initiate and determine the duration of thromboprophylaxis. 

The severity of infarction can vary and may be incidentally diagnosed in asymptomatic patients. Early diagnosis and treatment for infarction are critical for potentially salvaging the allograft. Given the predisposing risk of thrombotic diseases, it was proposed that abdominal CT scans should be considered in COVID-19 patients even with mild abdominal symptoms [21]. Retrospectively, elevated thrombotic and inflammatory tests in our case may also be utilized as surrogate markers for the propensity to develop thrombotic events. 

Generally, salvaging the infarcted areas of the allograft may be achieved by interventions including percutaneous endovascular or thrombolytic therapies. However, a small-sized allograft and pale-colored kidney indicate a remote onset of allograft injury from infarction, which was unlikely to benefit from the interventions. 

**Figure 6.**
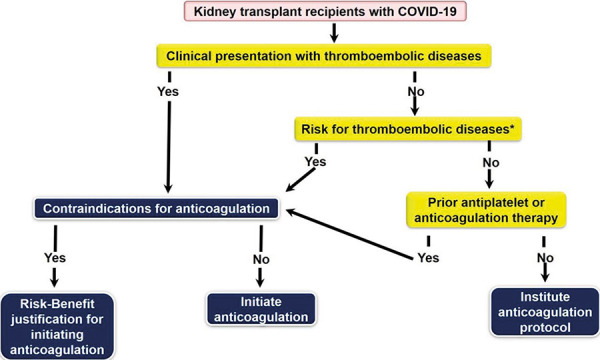
Proposed algorithm for anticoagulation management in kidney transplant recipients with COVID-19 by considering 3 main factors: clinical presentation suggesting thrombosis or confirmed diagnosis of thromboembolic diseases; patient risk factors for thromboembolic disease including traditional risk factors and risk for endothelial dysfunction or injury; and anticoagulation or antiplatelet prophylaxis or therapy received prior to having COVID-19. *Rraditional thromboembolic risk factors and possible additional risk factors such as previous vascular dysfunction, injury, or intervention.

Apart from traditional risk factors for thromboembolic diseases, TRAS ± TRAS intervention in kidney transplant recipients is also another risk factor that may increase the risk of thrombosis and then kidney allograft infarct. Although there is no evidence to make this conclusion, possible endothelial dysfunction-related to TRAS and previous vascular intervention may be a mechanism that contributes to an increased risk for transplant renal artery thrombosis and allograft infraction. Therefore, we propose a simplified protocol for anticoagulation in kidney transplant recipients with COVID-19 by taking three main factors into consideration including 1) clinical presentation suggesting thrombosis or confirmed diagnosis of thromboembolic diseases, 2) patient risk factors for thromboembolic diseases including traditional risk factors and risk for endothelial dysfunction or injury, and 3) anticoagulation or antiplatelet prophylaxis or therapy received prior to having COVID-19 (Figure 6). 

In conclusion, with an ongoing COVID-19 surge, transplant recipients continue to be at risk for COVID-19 with potentially serious complications. Emerging arterial and venous thrombotic complications related to SARS-CoV-2 and underlying immunological milieu can potentiate the risk of thrombosis. While there is insufficient evidence to conclude whether a history of TRAS increases the risk of thrombotic risk in the absence of negative imaging, uncertain but plausible endothelial dysfunction associated with TRAS and previous vascular intervention as well as elevated biomarkers of thrombosis particularly in patients with other high thrombotic risks may increase the risk for allograft infraction. Monitoring the biomarkers, serial allograft imaging, and appropriate thromboprophylaxis may be considered in transplant recipients. Although elevated biomarkers in COVID-19 are associated with overall morbidity and mortality outcomes, further high-quality evidence is required to guide anticoagulation therapy beyond current standard clinical practice. 

## Acknowledgment 

Authors appreciate our patient for the knowledge and experience we gain from clinical care and research. 

## Important disclosure 

KKZ serves as a physician in a US Department of Veterans Affairs medical centers with or without compensation or is a part- or full-time employee of a US Department of Veterans Affairs medical center. Opinions expressed in this paper are those of the authors and do not represent the official opinion of the US Department of Veterans Affairs. 

## Funding 

Supported by research grants from the National Institute of Diabetes, Digestive and Kidney Disease of the National Institutes of Health K24-DK091419, and philanthropic grants from Mr. Louis Chang and Dr. Joseph Lee. 

## Conflict of interest 

KKZ has received honoraria and/or grants from Abbott, Abbvie, Alexion, Amgen, DaVita, Fresenius, Genzyme, Keryx, Otsuka, Shire, Rockwell, and Vifor, the manufacturers of drugs or devices and/or providers of services for CKD patients. JEZ is a consultant for Leica Biosystems. ET, DCD, AJF, HI, RAL, AES, TF, JG, NK, and UGR have no relevant financial or non-financial interests to disclose. 

**Table 1.** Clinical characteristics related to COVID-19 of a previously published case of kidney allograft infarction and our case.

	Xu et al. [3] June 2020	Our case
Age/Gender	46/Male	33/Female
Cause of ESKD	T1DM	T1DM
Transplant	Kidney and pancreas transplantation / 13 years	0-A-B-DR mismatched antigen DDKT / 6.5 months
Immunosuppression	FK, MMF, and Pred	CsA, MPS, and Pred
Co-morbidities	Overweight (BMI 26.6 kg/m^2^)	Obesity (BMI 31.40 kg/m^2^) TRAS
The onset of COVID-19 until admission / Symptoms	14 days / nausea, diarrhea, weakness, worsening cough, and hypoxemia requiring O_2_	5 days / headaches, cough, fever, body aches, diarrhea, blood per rectum
COVID-19 diagnosis	a qualitative polymerase chain reaction test (Seegene Allplex 2019-nCoV Assay)	Nasopharyngeal swab by rtPCR
COVID-19 therapy	Lopinavir/ritonavir HQ and AZA 5 days and cefuroxime 7 days FK was held due to lopinavir/ritonavir administration, but MMF and prednisone were continued at the same doses.	MPS was initially held; then CsA was discontinued Remdesivir Dexamethasone Convalescence plasma
Thrombotic risks	Diabetes	Diabetes Hypertension Hyperlipidemia Obesity Hyperhomocysteinemia
Thromboembolic risks	None	New-onset atrial fibrillation
Thromboembolism prophylaxis / thromboembolic therapy	Enoxaparin 40 mg s.c. every 12 hours/ Enoxaparin 80 mg s.c. every 12 hours A plan to switch to apixaban 5 mg twice daily for ≥ 3 months	Heparin drip
Onset of renal infarction after COVID-19 diagnosis / Symptoms	26 days / Sharp LLQ pain 1 day	Hospital day 9 / Incidentally around from abdominal CT scan
Inflammatory biomarkers	Elevated WBC 27.4 (Ref 3.5 – 10.5 × 10^9^/L) Lymphocyte 0.8 (Ref 0.8 – 3.5 × 10 /L) Platelet 105 – 281 × 10^3^/µL (Ref: 130 – 380 × 10^9^/L)) INR 1.05 – 1.83 c-reactive protein 39.5 mg/L (Ref: ≤ 1 0) High-sensitive troponin I Procalcitonin D-dimer 744 µg/L (Ref: ≤ 500) Fibrinogen 7.9 g/L (Ref: 190 – 450) Ferritin 699: µg/L (Ref: 30 – 400) Interleukin-6 11.9 pg/mL (Ref: ≤ 2)	Elevated WBC 3.9 – 39.8 x 10^3^/µL(Ref: 4 – 10.5) Lymphocyte 0.1 – 0.6 × 10^3^/µL (Ref: 0.9 – 3.3) Platelet 105 – 281 × 10^3^/µL (Ref: 150-400) INR 1.05 – 1.83 Lactate dehydrogenase (Figure 4) hsCRP (Figure 4) hsTrop-I (Figure 4) Procalcitonin (Figure 4) D-dimer (Figure 4) Fibrinogen 778 mg/dL (Ref: 184- 419) Ferritin 501 ng/mL (Ref: 10 - 107) Interleukin-6 11.9 pg/mL (Ref: ≤ 2)
Hypercoagulable work-up	Negative Negative antiphospholipid antibody panel (anti-β_2_-glycoprotein 1 and anticardiolipin IgG/IgM antibodies, lupus anticoagulant) Absent Factor V Leiden and prothrombin gene mutations Normal antithrombin activity	Positive Elevated homocysteinemia 24 µmol/L (Ref: 5 – 15) Negatives Absent Factor V Leiden mutation Absent Prothrombin gene mutation Protein C 98% (Ref: 70 – 130) Protein S 55% (Ref: 57 – 113) Antithrombin III 102% (Ref: 80 – 120%) Anticardiolipin IgG < 9 GPL (Ref: 0 – 14) Anticardiolipin IgM < 9.4 MPL (Ref: 0 – 12.4) Negative Lupus antigoaculantation
Imaging findings	Kidney U/S: Decreased blood flow to the lower pole CT: A new cortical hypodensity in the lower pole LE U/S: No DVT EKG: NSR	Abdominal / Pelvic CT scan w/wo contrast: multiple kidney allograft infarcts Transplant kidney U/S with color Doppler: No flow in the mid and upper pole of transplant kidney, with punctate air densities, concerning infarction with gangrene. The lower pole parenchyma demonstrates a low resistive waveform. CTA: No evidence of pulmonary embolism
Outcomes	Cr 1.36 – 1.66 mg/dL Discharged home on day 19 after the onset of COVID-19 symptoms Readmission on day 26 after the onset of COVID-19 symptoms and was diagnosed with acute kidney allograft infarct.	Oliguric AKI requiring CVVHDF Acute hypoxemic respiratory failure requiring intubation New-onset atrial fibrillation Ventricular fibrillation and cardiac arrest Transplant kidney allograft nephrectomy. Pathology revealed diffuse thrombosis within glomeruli and many arteries/arterioles with marked cortical infarction. Reinitiating chronic intermittent hemodialysis

CsA = cyclosporine; AZA = azithromycin; BMI = body mass index; COVID-19 = Coronavirus disease 2019; Cr = creatinine; CT = computed tomography; DDKT = deceased donor kidney transplantation; DVT = deep venous thrombosis; EKG = electrocardiogram; ESKD = end-stage kidney disease; FK = tacrolimus; hsCRP = high-sensitive c-reactive protein; hsTrop-I = high-sensitive troponin; LLQ = left lower quadrant; MMF = mycophenolate mofetil; MPS = mycophenolate sodium; HQ = hydroxychloroquine; LE = lower extremity; NSR = normal sinus rhythm; rtPCR = a real-time reverse transcription polymerase chain reaction; sc = subcutaneously; TRAS = transplant renal artery stenosis; U/S = ultrasound
